# Unraveling TIME: CD8^+^ T cell– and CXCL11-driven endocrine resistance in breast cancer

**DOI:** 10.1172/JCI200923

**Published:** 2026-02-02

**Authors:** Tim Kong, Cynthia X. Ma

**Affiliations:** 1 Division of Oncology, Department of Medicine, Washington University School of Medicine, St. Louis, Missouri, USA.; 2 Department of Medicine, Weill Cornell Medicine, New York, New York, USA.

## Abstract

A major unmet need in estrogen receptor–positive (ER^+^) breast cancer is understanding the mechanisms that underlie resistance to endocrine therapy. Although accumulating evidence suggests an association between the tumor immune microenvironment (TIME) and endocrine response, the specific role of the TIME in mediating endocrine resistance remains unclear. In this issue of the *JCI*, Napolitano et al. analyzed tumor biopsies from patients with ER^+^ breast cancer and reported that endocrine-resistant tumors exhibited heightened CD8^+^ T cell infiltration and activation of the CXCL11 — CXCR3/-7 axis. Spatial and coculture analyses of these tumors demonstrated that the CD8^+^ T cell–associated chemokine CXCL11 drove estrogen-independent tumor growth. These findings identify an immune-mediated mechanism of endocrine resistance in breast cancer and identify CXCL11 as a potential biomarker and therapeutic vulnerability.

## Known endocrine resistance mechanisms in breast cancer

Estrogen receptor–positive (ER^+^) breast cancer accounts for the majority of breast cancer diagnoses and is predominantly categorized by luminal subtypes through transcriptional profiling (1), reflecting hormone-regulated gene expression. Endocrine resistance in ER^+^ breast cancer has traditionally been attributed to tumor-intrinsic mechanisms, including activation of growth factor signaling pathways such as PI3K/AKT and MAPK, dysregulated cell-cycle control (2), and epigenetic remodeling that sustains estrogen-independent transcriptional programs (3) as well as the acquired estrogen receptor α (ESR1) ligand–binding domain mutations under the pressure of endocrine therapy (4). These processes enable tumor cells to bypass estrogen dependence and limit the effectiveness of aromatase inhibitors and selective estrogen receptor degraders. However, these tumor cell–intrinsic pathways do not fully explain the heterogeneity of clinical resistance (2, 5).

## CD8^+^ T cells as unexpected partners in endocrine resistance

The tumor immune microenvironment (TIME) in breast cancer is highly heterogeneous and exerts a profound influence on tumor biology, treatment response, and clinical outcomes. It is composed of diverse immune cell populations, including tumor-infiltrating lymphocytes (TILs), whose abundance, phenotype, and functional state differ markedly across molecular tumor subtypes (6). CD8^+^ cytotoxic T lymphocytes (CTLs) are key components of the TIL population that recognize tumor antigens presented on MHC class I molecules and eliminate malignant cells primarily through the release of perforin and granzymes, as well as via death ligand–mediated apoptosis. High TIL levels, especially in triple-negative breast cancer (TNBC) and human epidermal growth factor receptor 2–positive (HER2^+^) breast cancers, are predictive biomarkers associated with improved prognosis (7, 8).

Although ER^+^ breast cancers generally display relatively lower levels of TILs (9, 10), accumulating evidence suggests that immune infiltration may modulate endocrine responsiveness. Notably, increased TILs and immune-related genes have been linked to a poor antiproliferative response to neoadjuvant aromatase inhibitor treatment (11, 12). Consistent with these observations, pooled analyses from the German Breast Cancer Group demonstrated that higher TIL levels were associated with worse survival in patients with luminal HER2^–^ tumors (10).

**Figure 1:**
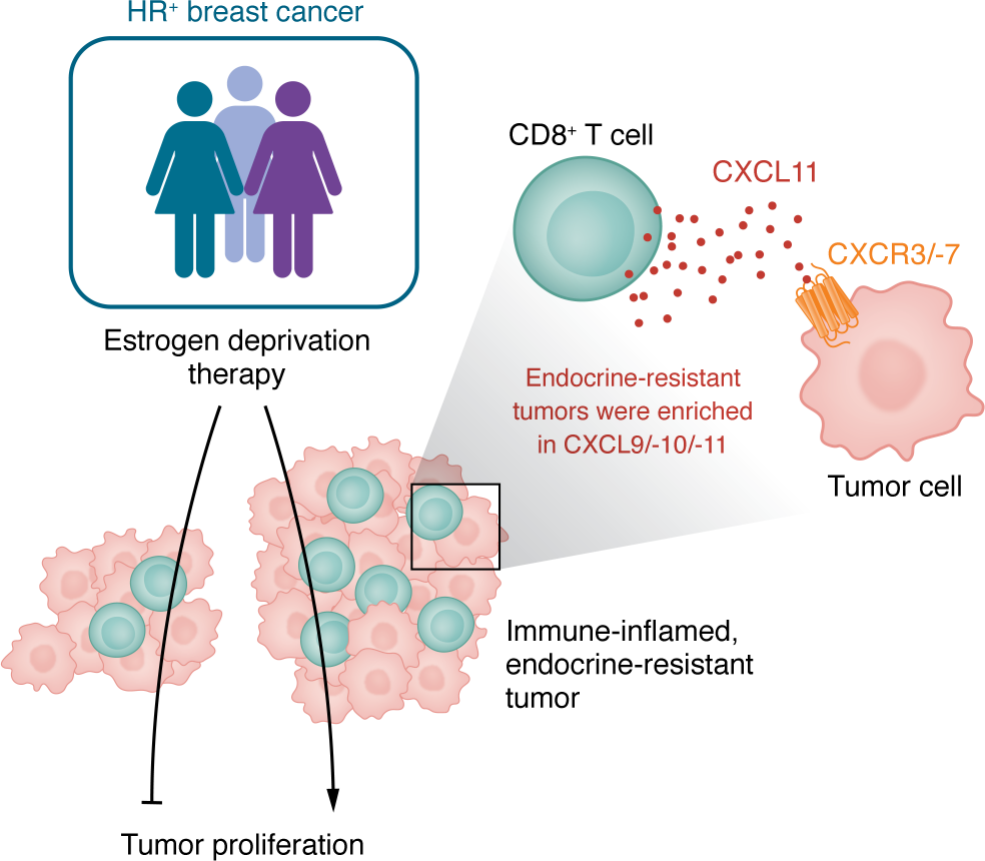
The CXCL9/-10/-11/CXCR3/-7 axis mediates resistance to estrogen deprivation in HR^+^ breast cancer. Napolitano et al. found that a subset of HR^+^ breast cancers resistant to estrogen suppression exhibited a TIME enriched with CD8^+^ T cells (13). The TIME in these tumors was also enriched in the chemokines CXCL9/-10/-11–CXCR3/-7 axis, and production of these chemokines by CD8^+^ T cells (and other immune cell populations) augmented tumor proliferation via their cognate receptors CXCR3/-7. Thus, rather than promoting effective antitumor immunity, the immune-inflamed environment in these endocrine-resistant tumors is subverted by CXCL11-mediated reprogramming to promote tumor growth.

Consistent with these findings, in this issue, Napolitano et al. (13) have demonstrated that tumors resistant to estrogen deprivation are not immune silent but instead exhibit an immune-inflamed phenotype (Figure 1). Through spatial transcriptomics and CIBERSORT-based cell population deconvolution of pre- and post-treatment biopsies from patients with hormone receptor^+^ breast cancer, the authors found that endocrine-resistant tumors harbored increased CD8^+^ T cell infiltration and showed upregulation of IFN-responsive and antigen-processing pathways in the TIME. Intriguingly, features that are typically associated with effective antitumor activity in other breast cancer subtypes were correlated with persistent tumor proliferation and endocrine resistance. This work continues to reframe the immune contexture of luminal tumors and suggests that immune infiltration does not necessarily equate to antitumor immunity.

Collectively, these findings point toward a subset of ER^+^ tumors that can be characterized as “immune-inflamed but endocrine-resistant,” raising the possibility that immune-tumor interactions may actively promote estrogen-independent growth and reflect a responsive, aggressive tumor adaptation or mechanism of endocrine resistance, rather than promoting effective antitumor immunity.

## CXCL11 connects CD8^+^ T cells to estrogen-independent growth

A central finding by Napolitano et al. was the selective enrichment of *CXCL9*, *CXCL10*, and especially *CXCL11* in estrogen deprivation–resistant tumors. These chemokines are canonically induced by IFN-γ to recruit immune cells, regulate their differentiation and activation, and propagate inflammation (14). CXCL11, in particular, is a robust chemoattractant of activated T cells into tumors (15, 16). In this study, spatial transcriptomics revealed that both immune and malignant compartments contributed to these chemokines, while coculture studies showed that CD8^+^ T cells promoted CXCL11 secretion. Functionally, recombinant CXCL11 induced estrogen-independent proliferation in HR^+^ breast cancer cell lines, activating the ERK and AKT pathways. Knockout of its cognate receptors CXCR3 or CXCR7 abolished this growth response, confirming a mechanistic requirement for CXCL11-driven signaling. Although CXCL11 is traditionally associated with antitumor immunity through the recruitment of CD8^+^ T cells, these findings recapitulate a paradoxical tumor-promoting role for CXCL11 in the context of estrogen suppression in ER^+^ breast cancer.

## Redefining the ER^+^ TIME

Napolitano et al. show that endocrine-resistant tumors exhibited a paradoxical immune-inflamed, yet tumor-permissive, microenvironment characterized by enriched CD8^+^ T cells, heightened IFN signaling, increased antigen-processing machinery, reduced Treg numbers, and elevated stromal TIL scores. Rather than initiating tumor clearance, this immune milieu appeared to support estrogen-independent growth, in part through CXCL11-mediated transcriptional reprogramming. These observations argue for reframing ER^+^ tumors not as immunologically “cold,” but as a subset with immune-subverted microenvironments.

## Clinical implications and opportunities

The findings by Napolitano et al. have several important clinical and translational implications. From a biomarker perspective, CXCL11 levels, either alone or as part of a CXCL9/-10/-11 chemokine signature, may serve as an early indicator of tumors unlikely to respond to endocrine therapy. Integrating such immune-based signatures with tumor cell–intrinsic biomarkers could refine patient stratification for endocrine therapy.

Therapeutically, the CXCL11 – CXCR3/-7 axis emerges as a potentially targetable pathway in ER^+^ breast cancer. While currently there are no clinical neutralizing antibodies against CXCL11, agents designed to block CXCR3 or CXCR7 signaling, or to modulate upstream IFN signaling cascades, could theoretically interrupt the proproliferative chemokine signaling initiated by CD8^+^ T cell–associated CXCL11. Small molecular inhibitors of CXCL3 such as AMG487 have been shown to inhibit lung metastasis in the murine 66.1 cancer model but lacked efficacy in reducing local tumor burden or enhancing animal survival (17). Further investigations and development of such interventions may restore endocrine sensitivity or prevent the development of estrogen-independent growth, offering a new direction for overcoming immune-mediated resistance mechanisms.

Importantly, the same tumor immune features that drive endocrine resistance, such as CD8^+^ infiltration, antigen presentation, and IFN activation, may also prime these tumors for response to immunotherapy. In this context, immune-inflamed ER^+^ tumors might paradoxically represent the subset most likely to benefit from checkpoint blockade, as suggested by encouraging emerging clinical data in high-risk breast cancer showing an increased pathological complete response with the addition of anti–PD-1 inhibitors to chemotherapy (18, 19). These insights support future clinical trials exploring rational combinations of endocrine therapy with immunomodulatory agents.

In parallel, advances in spatial transcriptomics provide an unprecedented opportunity to characterize the TIME with high spatial resolution. The spatial organization of immune cells relative to the tumor epithelium may predict early endocrine responses or identify tumor cell subpopulations at risk for adaptive resistance. Incorporating such spatial biomarkers into neoadjuvant trial designs could enable earlier therapeutic intervention and personalized treatment adaptation.

Together, these findings redefine the clinical paradigm of luminal breast cancer. By demonstrating that CD8^+^ T cells can facilitate tumor persistence under estrogen deprivation through CXCL11 signaling, Napolitano et al. reveal an immune-endocrine interplay that broadens our understanding of resistance biology and opens new avenues for therapeutic innovation.

## Unanswered questions

Despite these advances, several important questions remain. The triggers of CXCL11 induction are not yet fully understood, raising the possibility that estrogen withdrawal itself, alteration of IFN-γ signaling, preexisting immune activation, or their interaction may stimulate chemokine upregulation. Additionally, although CD8^+^ T cells appear to be a prominent source of CXCL11, the relative contributions of other immune cell populations, such as macrophages or DCs, require further clarification to define the full cellular circuitry of this pathway in vivo. It also remains unclear how estrogen receptor signaling intersects with immune regulatory networks; coordinated regulation of IFN-responsive and estrogen-dependent transcriptional programs may underlie the emergence of immune-mediated endocrine resistance. Finally, whether CXCL11 can serve as a predictive biomarker for responsiveness to immune checkpoint inhibitors in ER^+^ breast cancer warrants prospective clinical validation.

## Conclusion

This work adds an immune dimension to endocrine resistance, complementing established tumor cell intrinsic drivers such as ESR1 mutations, MAPK/PI3K pathway activation, and cell-cycle dysregulation. By demonstrating that CD8^+^ T cells can facilitate tumor survival under estrogen deprivation, Napolitano et al. highlight the necessity of incorporating the TIME into future models of endocrine resistance. Targeting the CXCL11-driven immune-endocrine axis may unlock new therapeutic avenues for improving outcomes in ER^+^ breast cancer.

## Funding support

This work is the result of NIH funding, in whole or in part, and is subject to the NIH Public Access Policy. Through acceptance of this federal funding, the NIH has been given a right to make the work publicly available in PubMed Central.

- Siteman Cancer Center Grant (P30 CA91842, SCC, Eberlein).
- Barnes and Jewish and Siteman Cancer Center Investment Grant.
- NIH (1R01CA275904, to CXM).
- Susan G. Komen Leadership Award (to CXM).
- Breast Cancer Research Foundation (to CXM).
- Saint Louis Men’s Group Against Cancer (to CXM).

## Version 1 02/02/2026

Electronic publication

## References

[1] Perou CM (2000). Molecular portraits of human breast tumours. Nature.

[2] Hanker AB (2020). Overcoming endocrine resistance in breast cancer. Cancer Cell.

[3] Garcia-Martinez L (2021). Epigenetic mechanisms in breast cancer therapy and resistance. Nat Commun.

[4] Raheem F (2023). Metastatic ER^+^ breast cancer: mechanisms of resistance and future therapeutic approaches. Int J Mol Sci.

[5] Chien TJ (2021). A review of the endocrine resistance in hormone-positive breast cancer. Am J Cancer Res.

[6] Harris MA (2024). Towards targeting the breast cancer immune microenvironment. Nat Rev Cancer.

[7] Gao ZH (2020). Predictive and prognostic role of tumour-infiltrating lymphocytes in breast cancer patients with different molecular subtypes: a meta-analysis. BMC Cancer.

[8] Ali HR (2014). Association between CD8^+^ T-cell infiltration and breast cancer survival in 12,439 patients. Ann Oncol.

[9] Stanton SE (2016). Variation in the incidence and magnitude of tumor-infiltrating lymphocytes in breast cancer subtypes: a systematic review. JAMA Oncol.

[10] Denkert C (2018). Tumour-infiltrating lymphocytes and prognosis in different subtypes of breast cancer: a pooled analysis of 3771 patients treated with neoadjuvant therapy. Lancet Oncol.

[11] Dunbier et al (2013). Molecular profiling of aromatase inhibitor-treated postmenopausal breast tumors identifies immune-related correlates of resistance. Clin Cancer Res.

[12] Schuster EF et al (2023). Molecular profiling of aromatase inhibitor sensitive and resistant ER+HER2- postmenopausal breast cancers. Nat Commun.

[13] Napolitano F (2026). CD8^+^ T cells in the tumor microenvironment modulate the response to endocrine therapy in breast cancer. J Clin Invest.

[14] Tokunaga R (2018). CXCL9, CXCL10, CXCL11/CXCR3 axis for immune activation - a target for novel cancer therapy. Cancer Treat Rev.

[15] Cole KE (1998). Interferon-inducible T cell alpha chemoattractant (I-TAC): a novel non-ELR CXC chemokine with potent activity on activated T cells through selective high affinity binding to CXCR3. J Exp Med.

[16] Gao Q (2019). Cancer-cell-secreted CXCL11 promoted CD8^+^ T cells infiltration through docetaxel-induced-release of HMGB1 in NSCLC. J Immunother Cancer.

[17] Walser TC (2006). Antagonism of CXCR3 inhibits lung metastasis in a murine model of metastatic breast cancer. Cancer Res.

[18] Cardoso F (2025). Pembrolizumab and chemotherapy in high-risk, early-stage, ER^+^/HER2^-^ breast cancer: a randomized phase 3 trial. Nat Med.

[19] Loi S (2025). Neoadjuvant nivolumab and chemotherapy in early estrogen receptor-positive breast cancer: a randomized phase 3 trial. Nat Med.

